# Intramedullary Spinal Cord Metastases from Differentiated Thyroid Cancer, a Case Report

**DOI:** 10.3390/life12060863

**Published:** 2022-06-09

**Authors:** Fabio Volpe, Leandra Piscopo, Mariarosaria Manganelli, Maria Falzarano, Federica Volpicelli, Carmela Nappi, Massimo Imbriaco, Alberto Cuocolo, Michele Klain

**Affiliations:** Department of Advanced Biomedical Sciences, University of Naples, Federico II, 80138 Naples, Italy; fabiovolpe84@outlook.it (F.V.); lea-17-08@hotmail.it (L.P.); mariarosaria.manganelli.87@gmail.com (M.M.); maria.fal@hotmail.it (M.F.); federicavolpicelli93@gmail.com (F.V.); c.nappi@unina.it (C.N.); mimbriaco@hotmail.com (M.I.); cuocolo@unina.it (A.C.)

**Keywords:** differentiated thyroid carcinoma (DTC), radiometabolic therapy, hybrid imaging, radioiodine, drop metastasis

## Abstract

Intramedullary spinal cord metastases (ISCM) are uncommon metastases of the spinal cord. Magnetic resonance (MR) plays an important role in surgical planning when ISCM is suspected in the differential diagnosis. The incidence of ISCM is expected to increase due to the longer survival of cancer patients as well as the widespread use of MR in the diagnosis of neurological syndromes. The management of these patients is controversial because of the multiple clinical presentations and lack of controlled studies on the efficacy of different therapeutic approaches. Increased awareness of this rare entity may lead to an earlier diagnosis with novel imaging approaches at a stage when neurological deficits are reversible. A case of ISCM in a 49-year-old patient with differentiated thyroid cancer is reported.

## 1. Introduction

Intramedullary spinal cord metastases (ISCM) are uncommon secondary localizations of primary cancer in the spinal cord. ISCM detection in oncological patients is rare (0.1–0.4%) and represents a small part of intramedullary spinal cord tumors (1–3%) [1]. Before magnetic resonance (MR) was available, most cases of ISCM were identified after postmortem examination [2]. From literature data, it has been observed that further systemic metastases are already present at the moment of ISCM diagnosis [3]. Lumbar and thoracic regions and less frequently the cervical region are often reported as sites of a single metastasis [4,5]. The majority of patients presented brain metastases, but leptomeningeal carcinomatosis could be present [2,6]. In half of the cases, lung cancer is the most common ISCM-related tumor and small cell lung cancer is the most common subtype. Other than this, ISCM may be seen in breast cancer (13%), renal cancer (4%), melanomas (9%), and/or lymphomas (5%) [7,8,9,10,11]. In metastatic spinal tumors, the age of diagnosis is frequently from 50 to 60 years. It has been reported that males are more frequently affected than females [11]. Symptoms such as pain and myelopathy develop within 2 months and plegia may develop in 10–15% of cases [3]. Motor deficits, sphincter problems, and sensory disorders are very common [10,12]. MR plays an important role in surgical planning when ISCM is suspected in the differential diagnosis. The incidence of ISCM is expected to go on increasing due to the longer survival of cancer patients as well as the increased diagnostic sensitivity due to the widespread use of MRI in the evaluation of neurologic syndromes. We report a case of ISCM in a patient with differentiated thyroid cancer (DTC).

## 2. Case Presentation

A 49-year-old man with suspected differentiated thyroid cancer (DTC) underwent total thyroidectomy and cervical lymphadenectomy. The histopathological diagnosis confirmed papillary carcinoma with Hurtle cell component, infiltrating perithyroidal tissue, and lymph-vascular embolization (pT1bN1bMx). Molecular biology examination showed the presence of V600E mutation of BRAF gene (mutation c. 1799 T > A). Adjuvant radioactive iodine (RAI) therapy with 3700 MBq of ^131^I was administered. At the time of RAI administration, serum thyroglobulin was 61 ng/dL, and TSH was 48.9 UI/mL.

**Figure 1:**
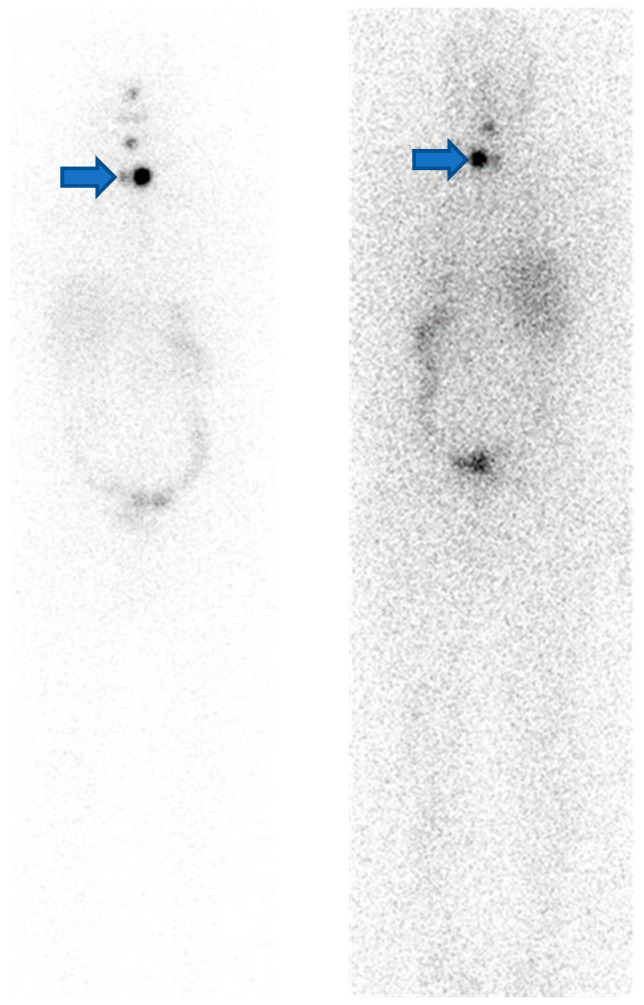
Whole-body scan after RAI therapy showing pathological uptake in the neck region (Blue arrows).

One week after RAI therapy, a planar whole-body scan (WBS) was performed (Figure 1) showing ^131^I accumulation in the neck region. Interestingly, at the same time, he developed an insidious onset of left-sided paresthesia. Bilateral neuropathic pain in the upper limbs was reported for a week. While proprioception was considered normal, a degree of pyramidal weakness of the right side, affecting both the upper and lower limbs, and a left impaired sensation at the T6 sensory level were discovered on neurological examination.

### 2.1. Pre-Surgical Intervention Imaging

**Figure 2:**
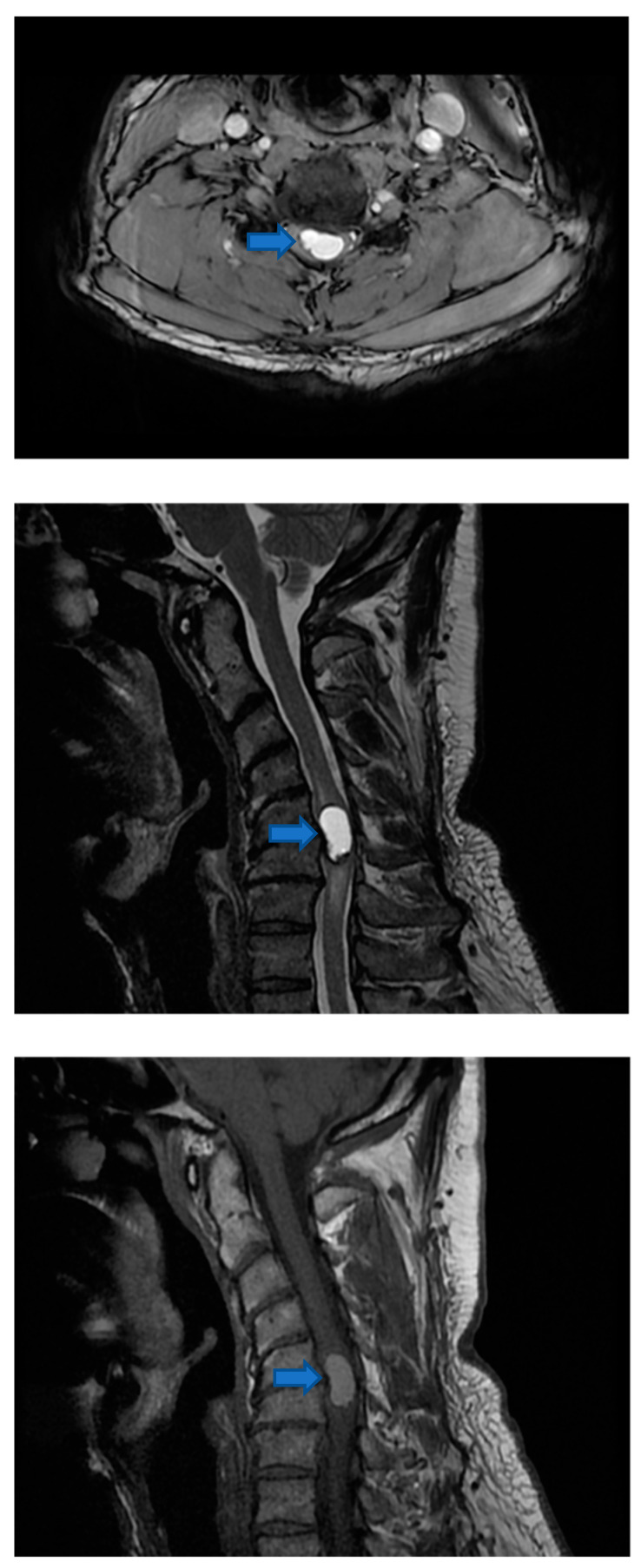
Pre-surgical MR (axial and sagittal T2 weighted images and sagittal post gadolinium T1 weighted image) detected an intramedullary lesion centered at the C5/C6 disc level (Blue arrows).

Due to neurological symptoms, the patient was referred to MR evaluation of the spine (Figure 2), which showed a well-circumscribed intramedullary 12 mm diameter lesion centered in the neck at the C5/C6 intersomatic disc level. The spinal cord was expanded at the same level, and the lesion showed post-contrast enhancement. Such a finding was associated with subtle surrounding T2 hyper intensity suggestive of vasogenic edema. There was a sign of an early syrinx within the cord, but neither necrosis nor cyst formation was identified.

### 2.2. Surgery

The patient was referred to neurosurgery for the lesion resection due to neurological impairment, and surprisingly the histopathological diagnosis on 12 mm tissue was a metastasis of papillary carcinoma (follicular variant [AE1/3, TTF-1, thyroglobulin positive, Ki67 10%]).

### 2.3. Post-Surgical Intervention Imaging

**Figure 3:**
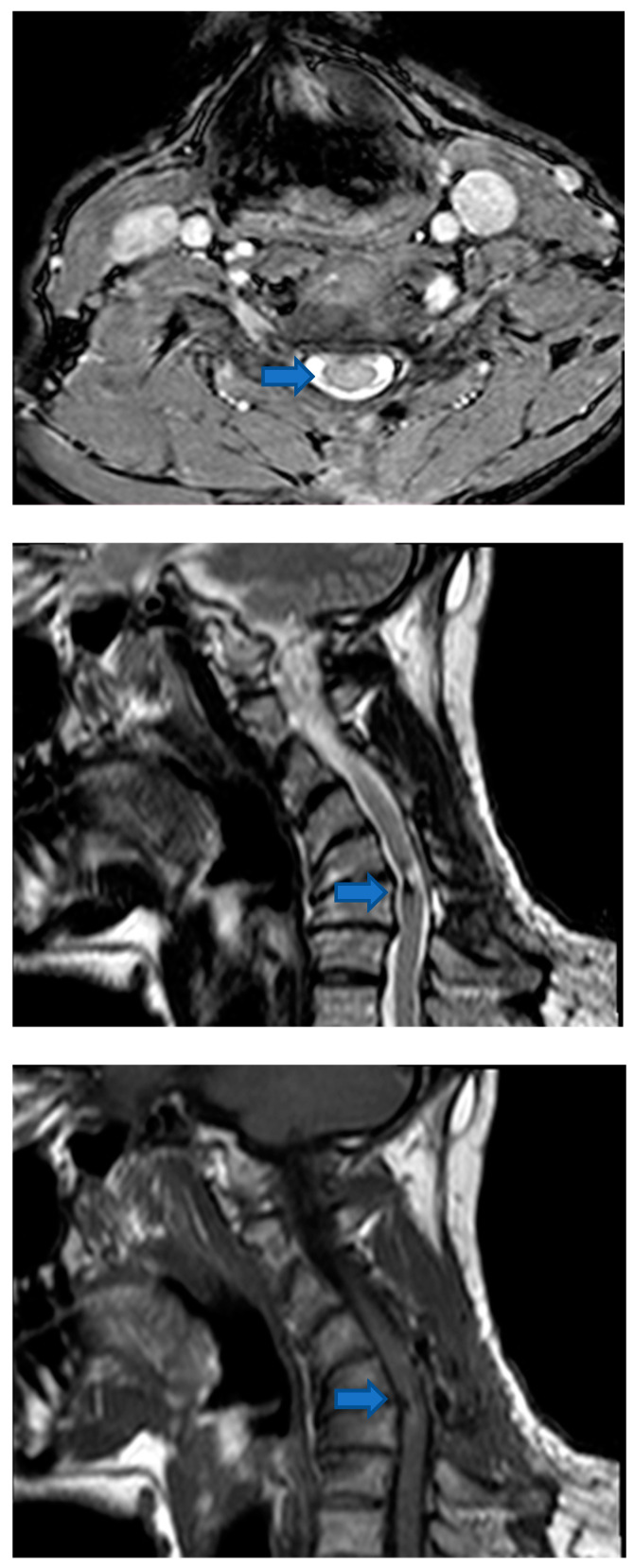
Post-surgical MR (axial and sagittal T2 weighted images and sagittal post gadolinium T1 weighted image) shows cervical spinal cord surgery signs at the C5/C6 level and normal thickness restoration (Blue arrows).

**Figure 4:**
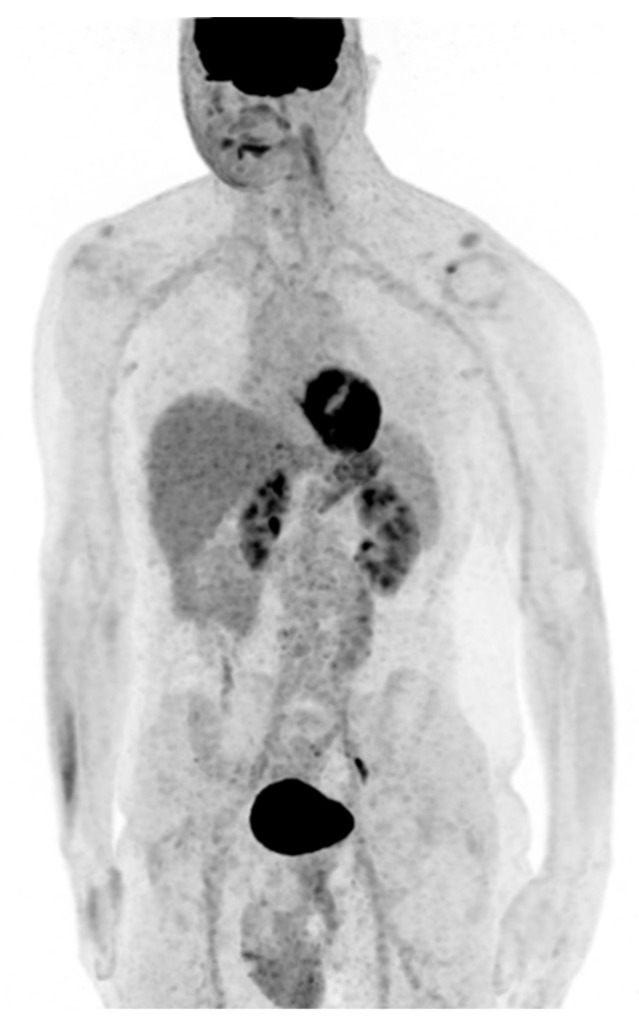
Post-surgical PET/CT with ^18^F-FDG does not show pathological uptake.

Post-intervention MR showed cervical spinal cord surgery signs at C5/C6 level and normal thickness restoration (Figure 3). The remaining neural axis was considered negative for the presence of other lesions. He was referred to a subsequent ^18^F-fluorodeoxyglucose (FDG) positron emission tomography (PET)/computed tomography (CT) study of the chest, abdomen, and pelvis that demonstrated no other secondary localizations (Figure 4).

Serum thyroglobulin was 0.2 ng/dL and TSH was 0.05 UI/mL under tyrosine-suppressive therapy.

## 3. Discussion

DTC is a large and heterogeneous family, including papillary or follicular types with a good prognosis and anaplastic thyroid cancer with a severe prognosis [13]. Distant metastasis from DTC is often correlated with primary tumor de-differentiation and with the capacity to leave the primary localization through local infiltration and via the lymphoid or hematic system [14,15,16,17]. Several genes are involved, including BRAF, NRAS, HRAS, PIK3CA, TERT, and AKT1 [18,19,20]. These oncogene mutations can provoke a radical change in thyroidal cancer cell membrane protein expression and enzyme functionality. For instance, the critical role of BRAF mutation V600E in DTC cell RAI uptake loss [21,22,23] but also in mitochondrial metabolism upregulation [24] has been reported. The ISCM-specific biochemical mechanism is not clearly known. As in other metastatic cancer models, it could involve membrane ligands, such as the TSH receptor [25], and extracellular matrix remodeling and degradation [26,27]. In particular, exosomal integrins and a compatible central nervous system microenvironment can drive metastatic cancer-specific trophism, including the central nervous system [28,29]. We reported a case of ISCM in a patient with DTC. Although WBS showed uptake in the neck area, the two-dimensional planar acquisition was not able to discriminate the exact level of the ^131^I uptake. In this case report, MR imaging guided by characteristic symptoms was able to accurately identify the lesion in the neck area.

This case highlights the role of tomographic morphological imaging in the accurate localization of extrathyroidal tumor foci. MR is historically the choice for neck cancer imaging [30,31] thanks to high local contrast and resolution. Not only MR imaging but also integrated approaches such as PET/RM, PET/CT or single-photon emission computed tomography (SPECT)/CT (17) could have a diagnostic role. ^18^F-FDG PET/RM has been proven to not have a clear diagnostic advantage over ^18^F-FDG PET/CT in the neck area [32,33].

It should be noted that although planar WBS was able to identify an RAI avid area in the neck region, the lack of morphological data did not allow accurate identification of metastatic localization, while a post ^131^I therapy SPECT/CT should be performed after RAI and can elucidate otherwise cryptic findings at a planar scan, detecting hidden metastasis, especially in patients with high serum thyroglobulin levels [34,35,36,37]. Metabolic hybrid imaging can also have a role in risk stratification for DTC patients just after surgery and before RAI administration [38]. MR may have a role in further definition and characterization of single lesion anatomical relationships, especially in preparing for surgery.

ISCM from primary DTC is a rare entity [12,39]. The diagnosis and treatment can be guided by CT and CT myelography but along with improvement in MR techniques, differential diagnosis rates are increasing [3,40]. High rates of mortality and morbidity are often associated with ISCM, but survival and local control rates are ameliorated in conjunction with microsurgical and oncological advancement. Decompression and tumor excision are the major keystones in the ISCM first approach. In order to decrease the tumor burden and let the neural parenchyma expand, sometimes adjuvant systemic chemotherapy or local radiotherapy can provide an increased benefit [41]. In addition, some new techniques are emerging such as interstitial thermal therapy, radiofrequency ablation, cryoablation, and stereotactic radiosurgery [42,43]. In cases of cancer of unknown primary (CUP), primary cancer histology definition can be obtained by excision. Radiotherapy and radiosurgery are used as adjuvant to surgery to increase local control when the primary cancer is known or histologically defined; a systemic therapy approach can be chosen accordingly. ISCM management is a multidisciplinary approach and there is not a gold standard treatment modality. Sometimes, tumor metastasis can develop despite multimodal approaches and surgical excision may be necessary. In such cases, the most effective treatment is the surgical removal of the metastasis.

## 4. Conclusions

The incidence of ISCM is not frequent but, when cancer patients present with spinal cord neurological symptoms, it should be accounted for in the differential diagnosis. Final management approaches remain under debate due to their rarity and the scarcity of solid clinical data, often collected through case reports and short case series. Imaging has a primary role in diagnosis and hybrid, metabolic imaging can depict a precise whole-body evaluation of the cancer sites; in particular, post-therapy SPECT/TC of the neck region should be mandatory and inserted into the clinical routine. Novel integrated approaches should be the choice in DTC patients, especially if metastatic foci are suspected.
